# Time-Resolved Radiation-Induced Conductivity of Polyimide and Its Description Using the Multiple Trapping Formalism

**DOI:** 10.3390/polym11122061

**Published:** 2019-12-11

**Authors:** Andrey Tyutnev, Vladimir Saenko, Aleksei Zhadov, Evgenii Pozhidaev

**Affiliations:** National Research University Higher School of Economics, Moscow 101000, Russia; aptyutnev@yandex.ru (A.T.); exfaust@yandex.ru (A.Z.); epozhidaev@hse.ru (E.P.)

**Keywords:** polymers, irradiation by electrons, small-signal regime, dispersive transport, numerical calculations

## Abstract

Polymer dielectrics subjected to intense radiation fluxes exhibit a radiation-induced conductivity (RIC). Polyimide is a good dielectric with excellent mechanical and thermal properties featuring high radiation resistance currently widely used in the spacecraft industry. Its RIC has been extensively studied in several laboratories. The purpose of the present study is to make a direct measurement of the RIC for both pulsed and continuous irradiation using a current sensing technique, which is contrary to the indirect method employing a surface-potential decay technique that is now preferred by spacecraft charging engineers. Our experiments are done in a small-signal regime excluding any recombination and dose effects. In combination with existing computer codes, we managed to develop further the conventional multiple trapping formalism and the RIC theory based on it. The main idea is to supplement an exponential trap distribution responsible for a dominant dispersive carrier transport in polymers with a small concentration of inherent deep traps which may or may not have an energy distribution. In line with this reasoning, we propose a tentative set of RIC model parameters for polyimide that accounts for the observed experimental data. The findings and their implications are discussed in a broad context of previous studies.

## 1. Introduction

The radiation-induced conductivity (RIC) in insulators (polymers included) refers to an additional conductivity in excess of their dark one when subjected to intense radiation fluxes. RIC studies in polymers have a long and fortuitous history dating back to 1956 [1] and, from the very beginning, used an approach first suggested by Rose in 1953 [2], which later became known as a quasi-band multiple trapping (MT) formalism [3,4]. In it, charge carriers (electrons and holes) generated by an ionizing radiation emerge in a conducting state. Their lifetime in this state is only too short, due to the presence of numerous traps distributed exponentially in the binding energy. Trapped carriers can be thermally de-trapped into the conduction zone to be re-captured immediately again. This chain of events continues until a carrier recombines or exits a sample. Hence the name of this type of carrier transport in polymers—the multiple trapping model (MTM). The MTM successfully described such different phenomena as the current injection in solids [5], thermally stimulated currents (glow curves) in irradiated polymers [6], and the time of flight (TOF) results in photoconductive organic materials (molecularly doped polymers included) [7,8]. It was only natural to base the RIC theory on the MTM formalism. The famous Rose-Fowler-Vaisberg (RFV) model fully incorporated it from the very beginning in 1982 [9]. Four years later, we developed a powerful computer code to numerically solve the RFV equations relating to the step-function uniform irradiation of an infinite polymer slab with the carrier balance being governed by the bimolecular recombination only [10]. Subsequently, this code has been extended to the case of a finite slab with the carrier exit to electrodes controlling the process [11]. These numerical codes allowed to overcome the main limitation inherent to analytical solutions, including closed-form ones [12], which required that the dispersion parameter be was less than 0.5.

All this time, leading research groups (Gross et al. [13,14], and currently French researchers [15]) adhered to the two-trap RIC model whose parameters were retrieved from experiments which were not intended to directly measure the RIC itself (this last approach was again first proposed by Gross [16,17]). In this respect, one should remember Hughes as an eager proponent of advanced ideas that were intensively developed in the radiation chemistry of organic solids and in the area of the charge carrier transport in photoconductive polymers (see review [18]). In this context, the paper [19] coauthored by Hughes deserves special attention. Published in 1983, it went mostly unnoticed by the RIC community. However, recently it initiated a hot debate [20] which led to the extension of the conventional RFV model, which is analyzed in the present work. 

As our modified RFV model is only at the development stage, it needs a most accurate disposition of experimental details of data acquisition, their processing, and interpretation assisted by numerical calculations to find model parameters that fit the RIC results.

## 2. Materials, Methods, and Problem Formulation

### 2.1. Materials

For our studies, we used commercial films of Russian-made polyimide (trademark PM-1), which is, polypyromelliteimide containing proprietary additives. This polymer may be considered as an analog of Kapton (DuPont, Wilmington, DE, USA). We investigated its RIC for quite a long time (see [21]). PM-1 films had a thickness of 12 μm and samples cut from this film were 40 mm in diameter. Al electrodes (about 50 nm thick and 32 mm in diameter) were thermally evaporated on opposite sample sides in vacuum. 

### 2.2. Methods

**Figure 1:**
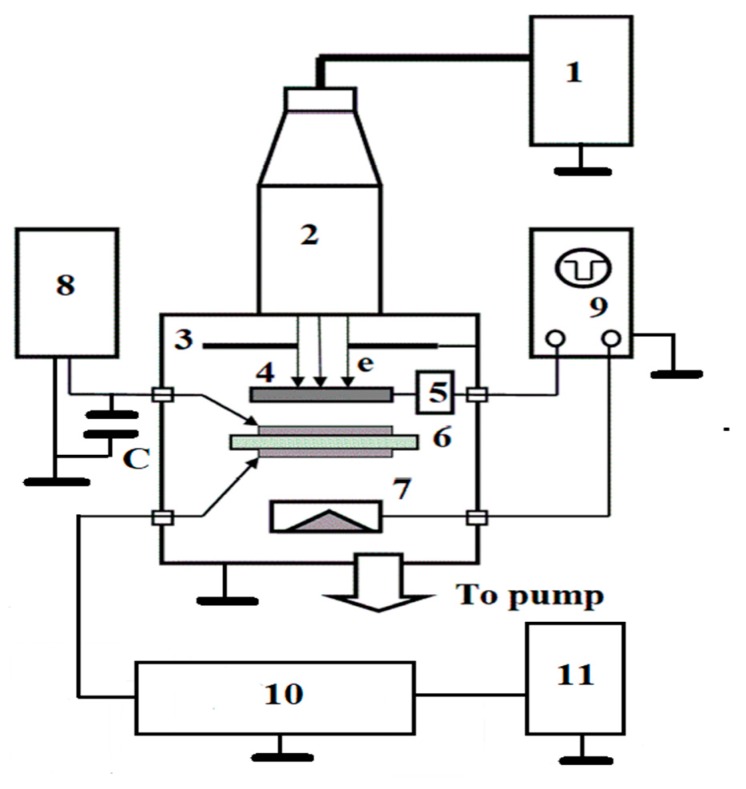
Schematic representation of the experimental setup for measuring polymer RIC in pulsed and continuous regimes. 1—high-voltage power supply; 2—electron gun; 3—electron beam collimator; 4—metallic shutter; 5—shutter control system; 6—test sample with evaporated Al electrodes; 7—Faraday cup; 8—DC voltage supply with an accumulative capacitor C and an electric circuit to put on and off the output voltage and control it; 9—double-beam Tektronix 3012B oscilloscope with a bandwidth of 300 MHz, 10—electronic block for measuring an analog RIC signal, amplifying and analog-to-digital converting and finally sending the ORIGIN current curve to printer 11.

Figure 1 gives a schematic representation of the experimental setup used in this study.

To irradiate the polymer samples, we employed 50-keV electrons supplied by an electron gun ELA-65. The experimental setup used in the present study was recently described in our latest works [20,21]. Here, we present a detailed description of it.

Beam electrons pass through a collimator (20 mm in diameter) bombarding a test sample to form an irradiation spot 30 mm in diameter. Between the collimator and the sample there is an Al shutter covered with an electroluminescent conducting paint. The shutter serves two purposes. First, it is used to not only to control an electron current after preliminarily calibrating it against a Faraday cup but also to visualize the current surface uniformity. Second, the shutter allows starting and terminating the continuous irradiation with an opening time 0.08 s. 

The electron current density was found to be constant to within 5% when measured over the entire irradiated surface. This finding was verified by registering an electron current by a shutter for some time and observing for current variations. They did not exceed the claimed 5% stability. The dose depth deposition was rather non-uniform in line with literature data (see for example [22]). Evidently, this is a problem for RIC assessment and methods to circumvent it, and this will be discussed below. 

The pulse duration was fixed at 20 μs and 1 ms, with typical rise and fall times at 0.7 and 10 μs, respectively. During the main part of the irradiation run (both pulsed and continuous) an electron current density was essentially constant, thus imitating a step-function dose rate profile. Furthermore, we used a small-signal irradiation regime as much as possible (violation of this rule is clearly noted). 

**Figure 2:**
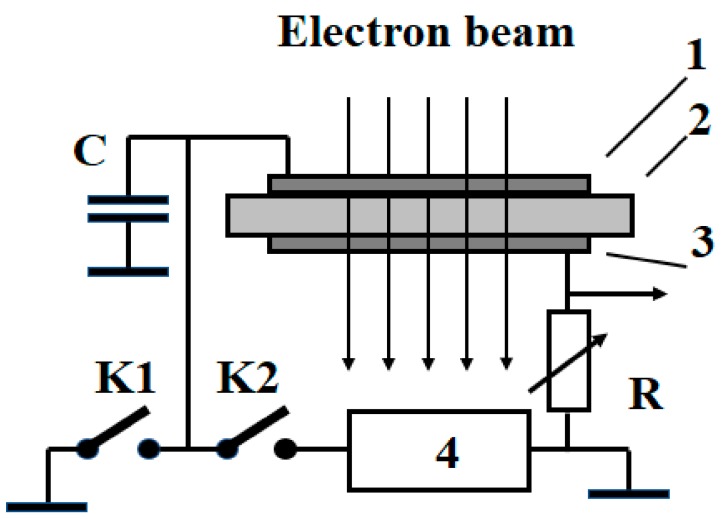
Schematic representation of the measuring setup. 1—potential electrode; 2—test sample; 3—measuring electrode; 4—voltage source. C is an accumulative capacitor as in Figure 1; K1 and K2 are switches (when one is open, the other is automatically closed); R is a load (measuring) resistor.

The test sample was a part of a series electrical circuit consisting of a voltage source (up to 1200 V) and a load resistor (Figure 2). The voltage drop over this resistor allowed determining the current passing through the sample. As in [20,21], to facilitate data reduction, this voltage drop was amplified, converted from the analog to digital format, put into a PC computer to be processed by the Origin program and finally stored for future use. When necessary, Origin files could be printed immediately.

But only part of this current defines the RIC proper, the other contributions come from the displacement (polarization) current and the radiation-driven current arising from specifics of the fast electron transport in a solid medium. The conventional description of the radiation-driven currents was given by Gross et al. [16] using a newly developed split Faraday cup technique. It was shown that some of the primary and secondary electrons stop inside a sample and cause a current to flow in the closed circuit even in the absence of the voltage source. It is important that the magnitude of the radiation-driven current is not affected by the applied voltage (in our experiments, less than 1200 V). 

#### Measuring Method

First, we put an electron gun into an operational condition for an intended experiment and determined the radiation-driven current $i_{rd}$ irradiating a sample with no applied voltage (switch K1 closed and switch K2 open) by a few pulses or alternatively, subjecting it to a continuous irradiation for a short period of time. 

Second, the shutter blocking the beam and switches being put in reversed positions (switch K1 open and switch K2 closed), the voltage was now applied for a few minutes, allowing a displacement (polarization) current to die out, which is easily achieved since PI is an excellent dielectric. This way, one gets rid of the displacement current even for continuous irradiations at the smallest dose rates used (see Figure 5, the star-marked curve) when conductivity under irradiation (∼ 10^−13^ Ω ^−1^ m^−1^) is much greater than the PI dark conductivity (about 10^−16^ Ω ^−1^m^−1^). After the depolarization procedure was over, we resumed an experimental run proper by putting off the shutter.

To find the current $i_{r}(t)$ directly associated with the RIC, one has to subtract algebraically the previously determined $i_{rd}$ from the measured total current $i_{rt}(t)$ passing through the poled sample under irradiation (as mentioned earlier, a displacement current is negligibly small). Once we know the RIC current $i_{r}$, the applied voltage V, the area of the irradiated spot S, and the film thickness L, the radiation-induced conductivity $\gamma_{r}$ can be assessed in a straightforward way:

(1) $$\gamma_{r}(t)=i_{r}(t)L/VS$$

The final aim of the experiment is to correlate the temporal dependence of $\gamma_{r}$ with the relevant dose rate $R_{0}$. To find it, we rely on our previous experimental and numerical (Monte-Carlo) simulations [21]. The best approach is to identify $R_{0}$ with the dose rate averaged over a sample thickness. For a current 100 nA measured by the shutter, it was estimated to be 190 Gy/s. Dose rate non-uniformity was ±30%. The minimum dose rate for continuous irradiation was 0.3 Gy/s and is limited by the beam current instability and the rf-noise in the measuring circuit. 

In time-of-flight (TOF) experiments, the notion of the radiation-induced conductivity $\gamma_{r}$ becomes meaningless, so one introduces a current density $j_{r}(t)=i_{r}(t)/S$ as the main output quantity.

Irradiations have been done in vacuum (approximately 10^−3^ Pa) at room temperature only. Most experiments used fresh (pristine) samples especially for continuous irradiations.

### 2.3. Problem Formulation

The interpretation of RIC results relies heavily on the nature of RIC phenomenon, multiple trapping formalism (dispersive transport of charge carriers), and the fundamental theories from the radiation chemistry of organic solids (an ion-pair radiolysis, the Onsager theory of the free carrier generation and the geminate conductivity) [23]. 

Because 50-keV electrons, like gamma- and X-rays, have a low energy transfer rate, they produce geminate ion pairs randomly in irradiated volume. For observation times exceeding some microseconds, geminate pairs have enough time to complete the initial recombination and emerge as separated electron-hole pairs in accordance with the Onsager theory [23]. Hence, their subsequent evolution (drift, diffusion and trapping) may be described by rate equations using appropriate kinetic coefficients for recombination, capture, and so forth. 

**Figure 3:**
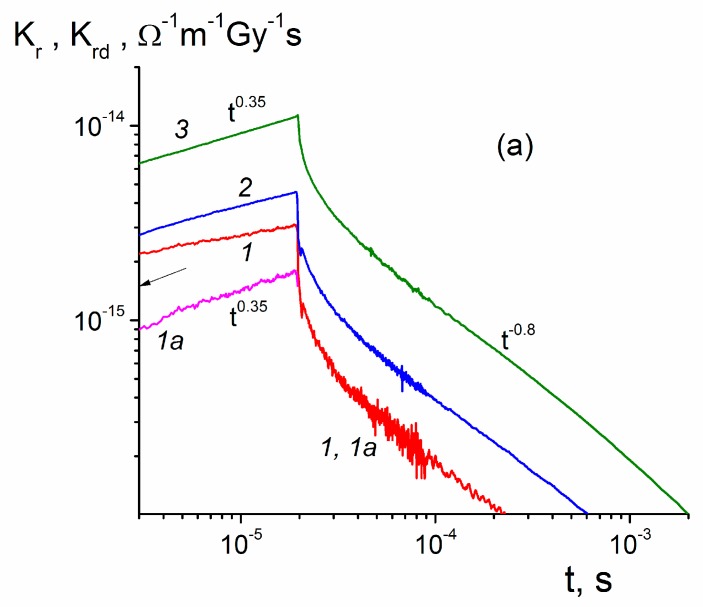

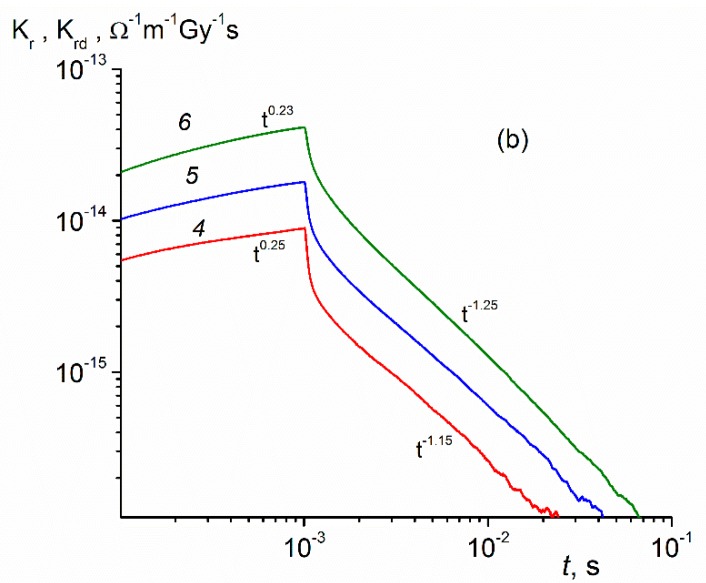
Normalized RIC ($K_{r}$) and RICd ($K_{rd}$) curves (*1–6*) and (*1a*), respectively. Pulse length 20 μs (Figure 3a, curves (*1–3*) and 1 ms (Figure 3b, curves (*4–6*). Electric field: 20 (1, 1a, 4), 40 (*2, 5*) and 80 V/μm (3, 6), small-signal irradiation regime. Normalized prompt conductivity $K_{p}=\gamma_{p}/R_{0}=$ 1.3 × 10^−15^ Ω ^−1^m^−1^Gy^−1^s is indicated by an arrow in Figure 3a.

It is known that RIC consists of two components [18]. Prompt one accounts for the carrier drift before trapping, while the delayed component is due to all carriers which experienced thermal de-trapping if only once: $\gamma_{r}=\gamma_{p}+\gamma_{d}$. At present, it is the general consensus that

(2) $$\gamma_{p}=K_{p}R_{0},$$

where $K_{p}$ is an empirical coefficient independent of an electric field and, partly, of temperature [18]. We estimated it using a triangular pulse with the full width at half maximum 1.5 μs: $K_{p}$ = 1.5 × 10^−15^ Ω ^−1^m^−1^Gy^−1^s. Test conditions are as follows: dose rate 2 × 10^5^ Gy/s, dose per pulse 0.35 Gy, electric field 5 V/μm) were enough to secure clear predominance of the prompt component over the delayed one. At longer pulses and stronger electric fields the opposite situation occurs. Hence, model parameters should be found by fitting experimental and numerical current curves relating to the RIC delayed components (below, RICd curves). The difference between RIC and RICd curves concerns not only their values but build-up shapes as well (compare curves (1) and (1a) in Figure 3a). 

As a prototype RIC model, we consider the modified RFV (RFVm) recently proposed in our paper [20]. The rate equations of this model are as follows:

(3) $$\partial \rho /\partial t=(N_{0}/\tau_{0})\left[M(E)-\rho \right]/M_{0}-\rho \nu_{0}\exp(-\frac{E}{kT})$$

(4) $$N=N_{0}+\int_{0}^{\infty }\rho dE$$

(5) $$dN/dt=g_{0}-k_{rec}N_{0}N_{}$$

It is seen that this is a purely time-dependent Cauchy-type problem. The first two equations are conventional in MT formalism, the last one accounts for the effect of a bimolecular recombination. Now, N is the total concentration of the mobile carriers (in our case, holes). Due to the charge neutrality, the total concentrations of holes and electrons (immobile carriers being recombination centers) are equal at any moment of time. $N_{0}$ is their concentration in the conduction zone where they have microscopic mobility $\mu_{0}$ and lifetime $\tau_{0}$. The energy trap distribution is M(E), with the total trap concentration being $M_{0}$ (note, trap energy is taken to be positive). The distribution function of trapped carriers is given by ρ(E). Furthermore, $\nu_{0}$ is the frequency factor, T—temperature, k—the Boltzmann constant, $g_{0}$—the generation rate of the separated electron-hole pairs (during irradiation constant) and $k_{rec}$—recombination constant. Now, we have to specify the trap distribution as a function of energy. The conventional RFV model uses in this case a simple exponential [18]

(6) $$M(E)=\frac{M_{0}}{E_{1}}\exp(-E/E_{1}),$$

where $E_{1}$ is, in fact, an average trap energy. Dispersion parameter $\alpha =kT/E_{1}$ controls RIC current shapes for step-function irradiation in a small-signal regime. As mentioned earlier, for α≤ 0.5 there are even closed-form expressions for these current shapes [12]. In a modified RFV model, the above trap distribution extends only to the separation energy $E_{s}$. For $E\geq E_{s}$ the distribution parameter of this exponential $E_{2}$ appreciably rises. Now, we have to deal with two dispersion parameters: $\alpha_{1}$ (the former α) and $\alpha_{2}=kT/E_{2}$. The idea of this separation is that each of the two trap fractions clearly identifies its contribution to the RIC, as suggested in [20]. An explicit form of M(E) is as follows

(7) $$M(E)=\frac{{\hat{M}}_{0}}{E_{1}}\exp(-E/E_{1}),\;E<E_{s}\;M(E)=\frac{{\hat{M}}_{0}}{E_{1}}\exp(-E_{s}/E_{1})\exp\left[(E-E_{s})/E_{2}\right],\;E\geq E_{s},$$

where ${\hat{M}}_{0}=M_{0}{\left[1+(\frac{E_{2}}{E_{1}}-1)\exp(-E_{s}/E_{1})\right]}^{-1}$. 

For reference, we indicate that the relative fraction of deep traps is equal to

(8) $$\eta =M_{2}/M_{0}=\frac{\left(E_{2}/E_{1})\exp(-E_{s}/E_{1}\right)}{1+(E_{2}/E_{1}-1)\exp(-E_{s}/E_{1})}$$

and for $\exp(-E_{s}/E_{1})\ll$ 1 we have

(9) $$\eta \approx \frac{E_{2}}{E_{1}}\exp(-E_{s}/E_{1}).$$

Here, $M_{2}$ is the concentration of deep traps with energies exceeding $E_{s}$. 

One note of caution. In the framework of the RFVm, the RIC prompt component ${\overset{⌢}{\gamma }}_{p}=g_{0}\mu_{0}\tau_{0}e$ (e is an electronic charge) is a field dependent quantity that duplicates the field dependence of $g_{0}$ contrary to the genuine RIC prompt conductivity which is field independent. 

## 3. Results

The most unambiguous information is provided by the RIC current curves relating to 20 μs square pulses (Figure 3a). We plot RIC and RICd data as conductivities reduced to a unit dose rate, namely $K_{r}$ and $K_{rd}$, in line with the reduced prompt conductivity (see Equation (2)). This procedure is quite legitimate for small-signal irradiations when both conductivities scale with the dose rate. However, unlike $K_{p}$, which is a constant, $K_{r}$ and $K_{rd}$ depend explicitly on time and parametrically on the electric field.

Let us concentrate on curves (*1*) and (*1a*). They serve to illustrate vividly the situation with the characterization of such curves. Note that they are plotted on a logarithmic scale to produce prolong straight lines whose slopes β=dlgK/dlgt (positive) near the pulse end or in an asymptotic decay region (usually longer than 3 or 4 pulse lengths) $\beta_{1}=-\beta$ (also positive) seem to be appropriate characterization parameters. We designate $\beta_{r}$ and $\beta_{d}$ for RIC and RICd curves, respectively. These slopes are indicated near curves as $t^{\beta_{r},\beta_{d}}$ or $t^{-\beta_{1}}$.

To characterize curves qualitatively, we indicate $K_{r}$ and $K_{rd}$ values at the pulse end. For example, for curve 1 $\beta_{r}$ is 0.16 and for curve 1a $\beta_{d}$ is 0.35 while $\beta_{1}$ is 0.8 for both curves with $K_{rd}$ (20 μs) = 1.7 × 10^−15^ Ω ^−1^m^−1^Gy^−1^s. The value $\beta_{d}$ = 0.35 for curve *1a* is surely to be associated with the dispersion parameter $\alpha_{1}$.

All these figures refer to an electric field 20 V/μm. It is remarkable that $\beta_{d}$ and $\beta_{1}$ stay unvaried as the field rises four times, which means that an important property is being captured by analyzing the RICd and not the total RIC producing unstable behavior. As expected, $K_{rd}$ (20 μs) steadily rises with field approximately as a power law $K_{rd}\propto F_{0}^{\delta }$ and δ≈ 1.1.

Figure 3b relating to 1 ms shows that these data are less exemplary, as $\beta_{1}$ is slightly unstable but power law $K_{rd}\propto F_{0}^{\delta }$ still holds. What is important is that $\beta_{d}$ drops to 0.24 for this pulse and this happens irrespective of an electric field. Values of $\beta_{1}$ exceeding unity (1.15 and 1.25), suggestive of the TOF effects, are in effect misleading (see later). 

**Figure 4:**
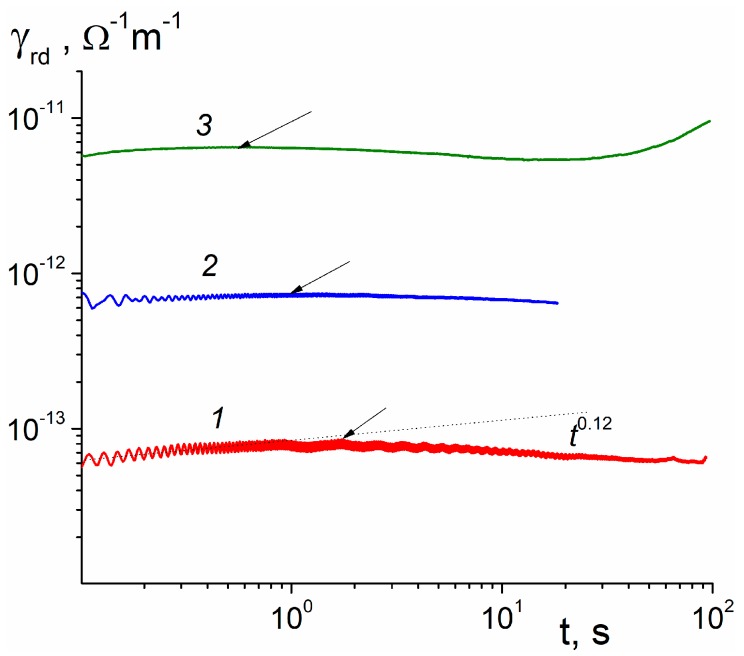
RICd curves for continuous irradiation with dose rate 1.7 (*1*), 17 (*2*), and 170 Gy/s (3). Arrows indicate maximum values of $\gamma_{rd}$. Electric field 40 V/μm, small-signal regime is operative only for curve (*1*) and only for t≤ 0.3 s.

Long-time, step-function irradiations are presently unavailable for the electron gun used. In this situation, we made continuous irradiations registering RIC currents from 0.1 s when a constant beam current was established (Figure 4). Curve *1* was taken at a low dose rate and produced $\gamma_{rd}$, rising as $t^{0.12}$ for t≤ 0.3 s. This finding shows that $\beta_{d}$ continues to decrease as the irradiation time increases.

At still longer times, RICd reaches a maximum at about 1.7 s. Increasing the dose rate (curves *2* and *3*) shifts this time to smaller times, the maximum value rises in accord with a power law $\gamma_{rd}\propto R_{0}^{\Delta }$ predicted by the conventional RFV model (see [18]). According to this model $\Delta ={\left(1+\alpha \right)}^{-1}$. In our case, α should be evidently replaced with $\alpha_{2}$. From Figure 4, we estimated an exponent Δ to be equal to 0.95 leading to $\alpha_{2}=$ 0.05. Curve *3* starts to rise even further after 20 s of irradiation but such a behavior should be ascribed to a dose effect (the so-called dose-modified RIC [21], whose nature is yet to be understood) and it happens in both PM-1 and Kapton. 

**Figure 5:**
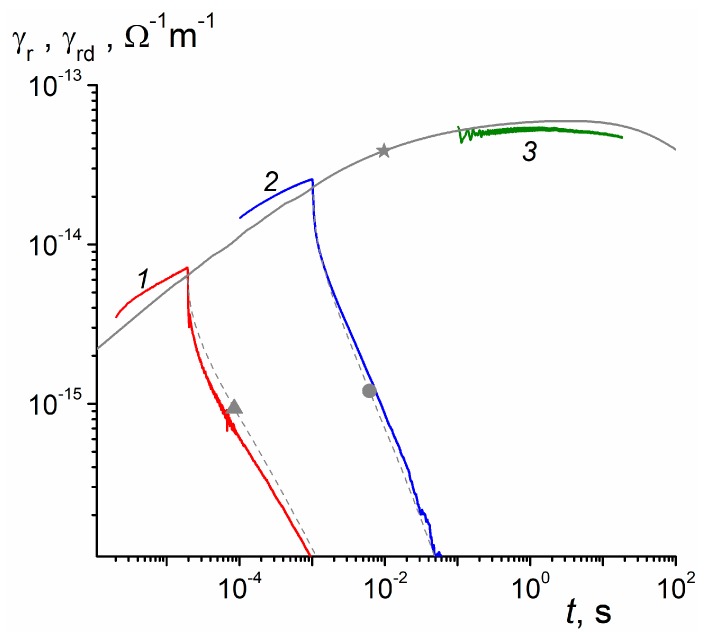
Experimental RIC (*1–3*) and computed (indicated by markers) RICd curves. Experimental dose rate 1.6 Gy/s and an equivalent generation rate $g_{0}$ used in numerical calculations is 10^20^ m^−3^s^−1^. Irradiation time 20 μm (*1*, triangle), 1 ms (*2*, circle), 30 s (*3*), and 100 s (star). RIC curves 1 and *2* are slightly scaled curves 2 and 5 in Figure 3. Electric field is 40 V/μm.

Based on our experimental data presented on Figure 3 and Figure 4 for an electric field 40 V/μm, we made a tentative attempt to describe RIC results with the RFVm model using the following model parameters (found by an iteration procedure): $\alpha_{1}$ = 0.4 ($E_{1}$ = 0.0625 eV), $\alpha_{2}$ = 0.01 ($E_{2}$ = 2.5 eV), $\nu_{0}$ = 10^9^ s^−1^, $\tau_{0}$ = 10^−11^ s, $\mu_{0}$ = 10^−6^ m^2^/V s, $k_{rec}$ = 2.4 × 10^−14^ m^3^s^−1^, $M_{0}$ = 10^26^ m^−3^, $E_{s}$ = 0.55 eV and η = 0.006 (according to Formulas (8) and (9)). Now, we have to specify a relationship between $R_{0}$ and $g_{0}$, allowing the comparison of computed and experimental data. For an electric field 40 V/μm, the radiation yield of free electron-hole pairs may be taken to be 0.7 per 100 eV of absorbed energy (like in polyethylene terephthalate used in [20]). Finally we get the relationship sought: $g_{0}$ (m^−3^ s^−1^) = 6.24 × 10^19^ $R_{0}$ (Gy/s) used in Figure 5, which shows the quality of fit of the numerical calculations to experimental results using the above set of RFVm model parameters.

**Figure 6:**
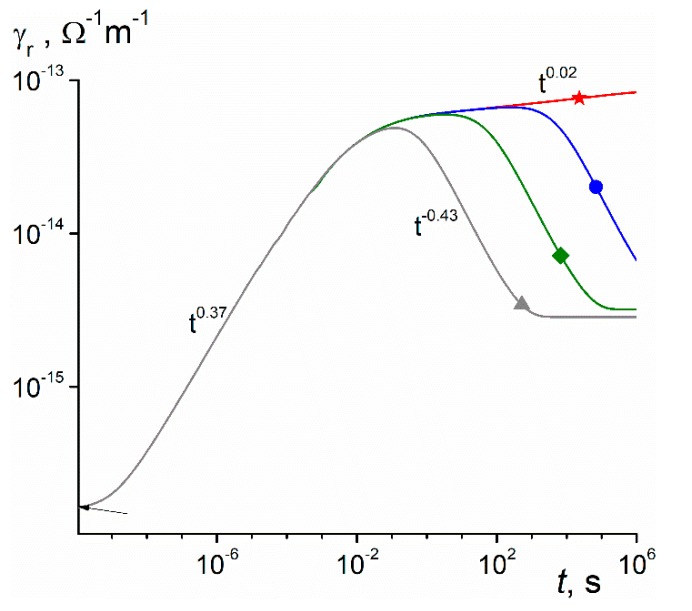
Computed RIC curves scaled by a factor ξ = 10^20^ m^−3^s^−1^/$g_{0}$ to make them to coincide at early times (t≤ 100 μs). Generation rate $g_{0}$ is 10^14^ (star), 10^18^ (circle), 10^20^ (diamond, unaffected by ξ), and 10^22^ m^−3^s^−1^ (triangle). The RFVm prompt conductivity is indicated by an arrow and is equal to 1.6 × 10^−16^ $\Omega^{-1}$ m^−1^ for diamond-marked curve. Irradiation time is 10^6^ s.

Computed RICd star-marked curve for the long-time irradiation well reproduces the build-up parts of RIC curves. Furthermore, it confirms that slope $\beta_{d}$ indeed falls off as irradiation length increases. At about 10 s, curve 4 reaches a maximum, indicating that the bimolecular recombination begins to play a decisive role. Certainly, it interferes with the curve shape already in the sub-second region where the small-signal regime begins to fail. To resolve this ambiguity, we extended numerical calculations of Figure 5 to dose rate extremes (Figure 6).

The form of presentation of computed curves in Figure 6 highlights all the important issues under debate. First, a star-marked curve allows to determine accurate values of $\beta_{d}$ at early times (from 1 to 100 μs when RICd clearly dominates over prompt conductivity). Its value, 0.37, is slightly smaller than $\alpha_{1}=$ 0.4 eV but larger than the experimental value 0.35, as Figure 3a shows. Second, the asymptotic value of conductivity rise $\gamma_{rd}\propto t^{0.02}$ (the expected law is $\gamma_{rd}\propto t^{\alpha_{2}}=t^{0.01}$) slightly fails. Third, a star-curve demonstrates limits of the small-signal irradiations (every curve for $g_{0}\leq$ 10^14^ m^−3^s^−1^ will simply fall on a star-curve). We see that increasing $g_{0}$ invariably places respective curves below a star-curve and more so the greater it is. Curve *3* is effectively curve *4* and now it becomes clear that the last curve should be treated as recombination free for times smaller than 0.3 s. Also, $\beta_{d}$ for a diamond-curve steadily falls with irradiation time constituting 0.25 at 1 ms, 0.1 at 0.1 s, 0.08 and 0.03 at 1 s, reaching the recombination limited maximum between 3 to 10 s when $\beta_{d}$ drops to zero. These data generally agree with the experimental results presented in Figure 3, Figure 4 and Figure 5.

Processing recombination affected curves in Figure 6 shows that the rad-ampere characteristic looks like an experimental one $\gamma_{rm}\propto g_{0}^{\Delta }$ (see Figure 4) with Δ = 0.95 in clear contradiction with the expected value (1 +$\alpha_{2}$)^−1^ = 0.99. However, to detect such small differences in Δ values at long irradiation times presents a real challenge to a researcher. Future work is needed to clarify this ambiguous issue.

## 4. Discussion

Radiation-induced conductivity of polymers was studied either under pulsed or continuous irradiations. Pulsed studies used bell-shaped pulses not well suited for a detailed kinetic analysis. Researchers had difficulty in separating contributions of the prompt and delayed RIC components. An empirical one- or two-trap model was used to interpret experimental results. No breakthrough information was obtained. Currently, these works are of historical interest only (see [24,25,26,27]). This early effort found logical conclusion in our works [9,28]. The last article dealt with pulsed (8, 40 ns and 0.3 ms) RIC data for a broad polymer list reporting values of $K_{p}$. Furthermore, for the first time, we proposed an RFV treatment of pulsed irradiations that introduces the notion of the initial effective mobility $\mu_{in}=\frac{\alpha }{1+\alpha }\mu_{0}\tau_{0}\nu_{0}$ [28]. In addition, we developed a simple analytic approach to estimate the frequency factor itself. Finally, using William’s approach [29] in combination with $\mu_{in}$, we suggested a simple estimation of the recombination time of the geminate electron-hole pairs that were later confirmed by numerical simulations [30,31]. Thus, our works [9,28,29,30,31] developed a methodology (both experimental and theoretical) for interpreting the RIC pulsed experiments.

As for continuous irradiations, it was a common practice to keep the dose rate constant, thus realizing a near step-function electron-beam (or an X-ray) profile. To avoid the dose effects, experiments were done only on fresh samples. The main information of these early studies was an exponent Δ in a power-like dependence of the steady-state conductivity on dose rate, which usually was less than 1 Gy/s [1] (a list of appropriate references may be found in [32]). Later RIC investigations using intense electron beams (10–1000 Gy/s) clearly demonstrated that there was no RIC steady state. Instead, there was a very slow approach to a maximum. Gross et al. [33] were first to report this result, relying on the two-trap model [33,34]. Our studies confirmed this finding but an explanation has been given using the RFV model [32]. Later, we discovered that the time of RIC maximum (current overshoot) may be substantially lengthened because the bimolecular recombination may become essentially retarded compared to the Langevin mechanism [35]. This phenomenon was called the non-Langevin recombination and was widely reported afterwards [36].

As already mentioned, a two-trap quasi-band model proved very popular in studies of the electron charging of polymer slabs with non-penetrating beams [16,17]. Recently, it found a new lease on life for the interpretation of charging polymer films (used or intended for use) in spacecrafts by electron beams closely imitating the spectra of plasma electrons in Earth orbits [15,37]. The ONERA researchers employed the surface potential decay method sometimes in the presence of fully penetrating electrons with a current density close to real values in orbit. Analyzing these data allows to find the RIC decay curves from which the two-trap model parameters can be retrieved [38,39,40]. In our opinion, this information should not be directly compared with RFV model parameters in view of discrepancies between RIC data found by potential-decay method and a direct RIC current measuring technique reported in [38]. The model of charge carrier transport in polymer slabs irradiated with non-penetrating electrons has been reviewed in [41].

A two-trap model suffers serious deficiencies if used for analysis of the RIC in polymers. It totally ignores the contribution of the prompt conductivity as well as the multiple trapping type of a charge carrier transport in disordered organics. Hence, this model should be viewed only as an engineering tool for fitting experimental data referring to electron charging phenomena in which the RIC plays an important but yet to be defined role. The RFV model is certainly a step forward in describing RIC in polymers. We applied it to make probe numerical calculations of polymer charging with non-penetrating electrons, as well as bulk charging by substorm electrons of the SCATHA geosynchronous orbit environment [42]. In this respect, one should mention yet another microscopic theory of RIC based on the α-, β-, and γ—relaxations (molecular motions) in polymers (see review by Khatipov [43]). Kinetic equations describing carrier transport are unnecessary cumbersome and lack physical transparency. As a result, it has not been accepted by the RIC community. Our attempt to include effects of molecular motions into the RFV model proved inconclusive as well [44].

After a ten year break devoted to the study of charge carrier transport in molecularly doped or photoconductive polymers (see [18] and our latest papers on the subject [45,46]), we turned our attention back to RIC in polymers [20,21], paying special attention to the small-signal step-function irradiations in both pulsed and continuous regimes [20]. In doing so, we relied heavily on our previous experience in the field. The real problems have started to emerge as soon as we compared pulsed and continuous irradiations of the same polymer samples under identical conditions using a small-signal regime.

The RFV model requires that under these conditions two fundamental relationships hold:

(10) $$\beta_{1}=(1-\alpha )$$

and

(11) $$\Delta ={\left(1+\alpha \right)}^{-1},$$

where Δ defines now a dose rate dependence of the maximum radiation-induced conductivity. Both relationships grossly fail in PM-1. Indeed, the dispersion parameter at early times (1 to 20 μs) is about 0.35 (see Figure 3a) which would require $\beta_{1}$ = 0.65, while its experimental value was between 0.8 and 1.1 (see Figure 3a,b). More to that, an approach of RIC to its maximum should follow a power law $\gamma_{rd}\propto t^{\beta_{d}}$ with $\beta_{d}=\alpha$ almost to a maximum itself (Figure 1 in [18]). Experimental value of Δ is 0.95 and according to Equation (11) α is expected to be 0.05, which differs appreciably from both $\alpha_{1}$ and $\alpha_{2}$.

In our previous publications [20,21], it has been shown that similar discrepancies have been found in Kapton and even more fragrant behavior has been observed in polyethylene terephthalate (PET) or polyethylene naphthalate (PEN). It is to reconcile pulsed and continuous irradiations in these commercial polymers that the RFVm model has been proposed in [20]. Nevertheless, there exists a group of polymers (photoconductive polyvinylcarbazole and molecularly doped polymers) including an ordinary polymer (low density polyethylene) that strictly follow the above RFV requirements [20]. Polymer assignment to the RFV or RFVm model depends critically on the comparison of pulsed and continuous irradiations under the conditions outlined above.

Future numerical work with the RFVm model analogous to that already done with the RFV model (see [18]) is highly needed to elaborate procedures producing one-valued model parameters with a clear physical meaning.

## 5. Conclusions

The combined pulsed and continuous irradiations of polyimide films (trademark PM-1) show that the conventional RFV model fails to describe RIC results consistently. For this purpose, we used the modified RFVm model proposed recently in our paper [20]. It replaces a simple exponential trap distribution of the conventional RFV model with an aggregate two-exponential one. This modification should not be confused with a two-trap model (now a model of choice in spacecraft charging community) with both traps having fixed energies. Finding the model parameters that fit the obtained RIC results was assisted by numerical calculations. A tentative set of the RFVm model parameters describing experimental data is proposed. Future work is needed to improve the accuracy of retrieving model parameters from the experiment, due to the discovered interconnection of the RFVm parameters requiring an application of the trial and error method that is contrary to a much more straightforward parameter selection of the conventional RFV model.

Our detailed analysis of the measuring method used to find the RIC in a polyimide PM-1 and develop the RFVm shows the importance of the proper accounting of the radiation-driven current and the RIC prompt component mostly overlooked in current investigations.
